# Dependency Network Analysis (D_EP_NA) Reveals Context Related Influence of Brain Network Nodes

**DOI:** 10.1038/srep27444

**Published:** 2016-06-07

**Authors:** Yael Jacob, Yonatan Winetraub, Gal Raz, Eti Ben-Simon, Hadas Okon-Singer, Keren Rosenberg-Katz, Talma Hendler, Eshel Ben-Jacob

**Affiliations:** 1 Sagol School of Neuroscience, Tel Aviv University, Tel Aviv, Israel; 2 School of Physics and Astronomy, Tel Aviv University, Tel Aviv, Israel; 3 Functional Brain Center, Wohl Institute for Advanced Imaging, Tel Aviv Sourasky Medical Center, Tel Aviv, Israel; 4 Biophysics Program, Stanford University, Stanford, CA, USA; 5 The Steve Tisch School of Film and Television, Tel Aviv University, Tel Aviv, Israel; 6 Sackler Faculty of Medicine, Tel Aviv University, Tel Aviv, Israel; 7 Department of Psychology, University of Haifa, Haifa, Israel; 8 Center for the study of Movement, Cognition, and Mobility, Department of Neurology, Tel Aviv Sourasky Medical Center, Tel Aviv, Israel; 9 The School of Psychological Sciences, Tel Aviv University, Tel Aviv, Israel; 10 Center for Theoretical Biological Physics, Rice University, Houston, TX, USA

## Abstract

Communication between and within brain regions is essential for information processing within functional networks. The current methods to determine the influence of one region on another are either based on temporal resolution, or require a predefined model for the connectivity direction. However these requirements are not always achieved, especially in fMRI studies, which have poor temporal resolution. We thus propose a new graph theory approach that focuses on the correlation influence between selected brain regions, entitled Dependency Network Analysis (D_EP_NA). Partial correlations are used to quantify the level of influence of each node during task performance. As a proof of concept, we conducted the D_EP_NA on simulated datasets and on two empirical motor and working memory fMRI tasks. The simulations revealed that the D_EP_NA correctly captures the network’s hierarchy of influence. Applying D_EP_NA to the functional tasks reveals the dynamics between specific nodes as would be expected from prior knowledge. To conclude, we demonstrate that D_EP_NA can capture the most influencing nodes in the network, as they emerge during specific cognitive processes. This ability opens a new horizon for example in delineating critical nodes for specific clinical interventions.

It has been long acknowledged that communication between and within brain regions is an essential element for effective network processing. More so, the influencing relationship between nodes in a network may point to regions that are most relevant for a specific task or state.

Several methods have been developed to estimate functional connectivity in fMRI, designed to search for subgroups of highly co-activated regions during “resting state”^1^. In order to discern such a functional relationship in different contexts, the seed region functional connectivity was further developed by the psychophysiological interactions (PPI) approach^2^. However, these methods provide the co-activation for one network’s node at a time, thus precluding an integrative measure of coupling between different nodes in the network. In an attempt to deal with this limitation, data-driven whole brain approach such as principal component analysis (PCA)^3^ and independent component analysis (ICA)^4^ have been used to extract distributed brain activation that contributes commonly to the explained variance in the signals. Yet, these methods are limited in addressing the influencing relationships among nodes of a given network during task performance^5^. For example, these methods can identify the motor network, but cannot determine the most influencing nodes during performance on a motor task. This type of information regarding nodes in the network is needed to better characterize its functional specificity^6^ and accurately target the most influencing nodes on task performance. Furthermore, it can provide better neural targeting for clinical interventions.

Effective connectivity methods^7^ were developed to assess the influences of nodes within a network. Currently there are two main approaches to address this issue: the first is dynamic causal modeling (DCM)^8^, which is based on a generic Bayesian probability model aiming to determine the direction of relations between nodes within a functional network. This method requires an a-priori model that delineates the directions of influence among the network’s nodes, and is thus limited when the tested network consists of more than a few nodes. The second method is Granger causality analysis^9^, in which temporal precedence is used to identify the direction of causality. While this method is data driven and not model based, it requires a relatively high temporal resolution, which is limited when using standard fMRI testing. Therefore, there is currently no satisfactory manner in which to determine the functional relations of a given network or set of networks during task performance.

In this work we aim to provide a computational approach to determine the level of influence of nodes within a network using a graph theory approach. Analysis methods based on graph theory were found reliable to detect complex network properties in fMRI including hierarchical organization of the network^10^,^11^,^12^. However, most fMRI studies to date have been based on measures of functional connectivity resulting in undirected graphs of brain networks^13^. Few studies have applied Granger causality on resting state fMRI data in order to construct a directed graph^14^,^15^, with these studies constrained by the temporal resolution required by the Granger methodology.

In order to provide directed graphs with minimal constrains we applied a new graph theory based approach for fMRI data analysis, entitled Dependency Network Analysis (D_EP_NA). This method was originally introduced for the study of financial data^16^,^17^, and has been extended and applied to other systems, such as the immune system^18^, and the study of semantic networks^19^. This method evaluates a node’s centrality within a given network according to its correlation influence; namely its effect (or contribution) on the correlations between all other pairs of nodes during task performance. Partial correlations between the time courses of the network’s nodes are used to quantify the node’s impact on the network. Simply put, the method provides each node with an *‘Influencing Degree’*, defined as the sum of influences of the node on all other nodes in the network, and *‘Influenced Degree’* as the sum of influences of all other nodes on it.

In a preliminary proof of concept study we demonstrate that the described approach successfully captures the true hierarchy of influence within a network. This will be presented both on simulated fMRI data and two empirical fMRI data during two task types; motor task of hand/leg moving and cognitive task of visual working memory.

## Results

### Dependency network analysis (D_EP_NA)

**Figure 1:**
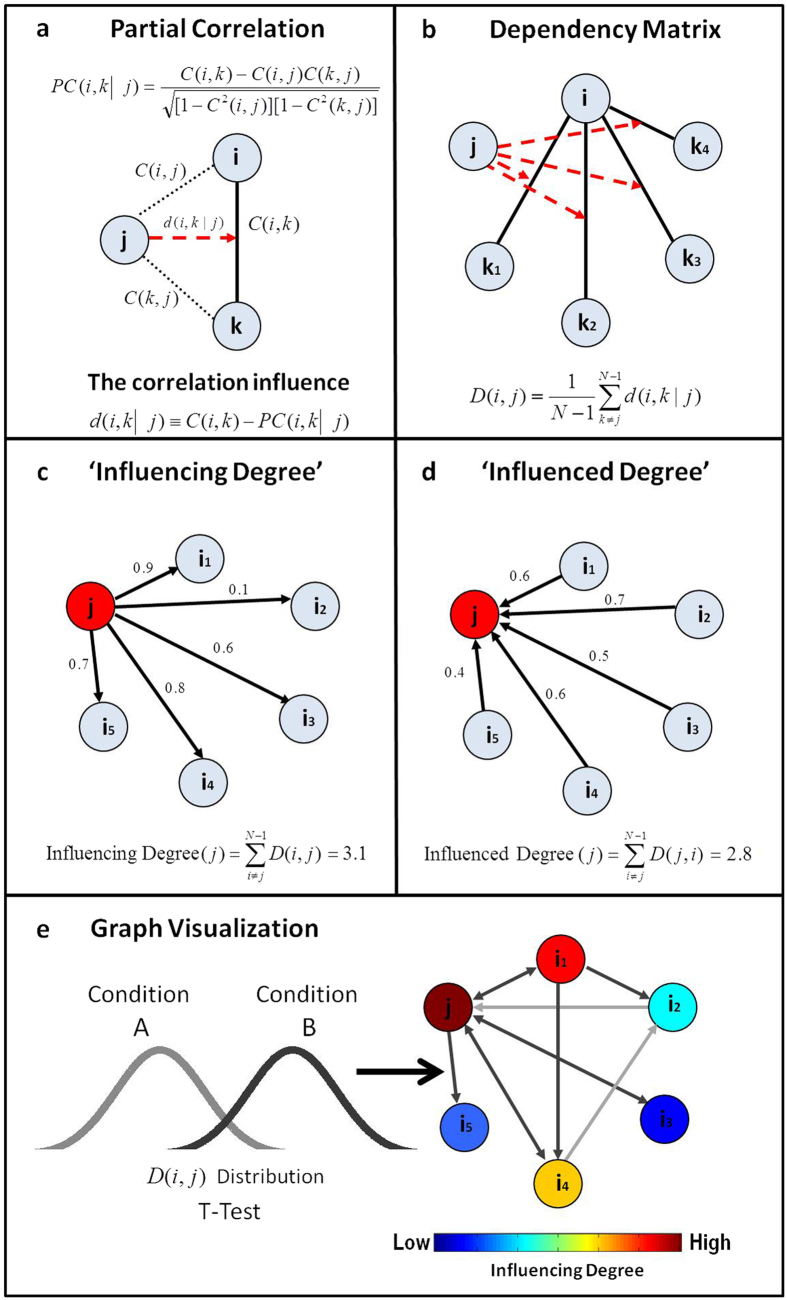
Dependency network analysis (D_EP_NA). **Step 1: (**a**) Partial correlation coefficient** – a statistical measure indicating how a third variable affects the correlation between two other variables. For example the partial correlation between nodes i and k with respect to a third node j - PC(i,k|j) defined in the equation. Where C (i,k), C (i,j) and C (k,j) are the ROI-ROI correlations. We then define the influence of j on the pair of elements i and k as the difference between the correlation and the partial correlation. Step 2: (**b**) Dependency Matrix– Next, we calculate the partial correlation effect for each ROI on all other pairwise correlations in the network. We define the total influence of node j on node i, D (i,j) as the average influence of node j on the correlations C (i,k), over all nodes k. The node dependencies define a dependency matrix D, whose (i,j) element is the influence of node j on node i. It is important to note that the dependency matrix D is nonsymmetrical since the influence of node j on node i is not equal to the influence of node i on node j. Step 3: (**c**) ‘Influencing Degree’– We then define the influences of node j as the sum of the influence D(i,j) of j on all other nodes i. The higher this measure the more this node influenced all other connections in the network. Step 4: (**d**) ‘Influenced Degree’– The dependency matrix is nonsymmetrical, therefore we can also sum all the influences (or dependencies) of all other nodes i in the network on node j D(j,i). The higher the influenced degree measure the more this node was influenced by all the other nodes in the network. Step 5: (**e**) Context related Graph Visualization – Each ROI is color-coded according to its influencing or influenced degrees. All pairwise ROIs with dependency elements D that are significantly different between two conditions (or groups) at the p < 0.001 level are plotted as edges. Each edge is color-coded according to the t-test sign as light or dark grey. The arrows represent the direction of influence.

In our method, the steps needed to indicate the network’s regions of interest (ROIs) *‘Influencing Degree’* or *‘Influenced Degree’* are (see Fig. 1); *(Step 1)* Compute partial correlations coefficient matrices. We then define the influence of node j on the pair of elements i and k as the difference between the correlation and the partial correlation. This quantity is large only when a significant fraction of the correlation between nodes i and k can be explained in terms of node j. (*Step 2)* Compute the dependency matrix. We calculate the partial correlation effect for each ROI (i.e. node) on all other pairwise correlations in the network. We define the total influence of node j on node i, D(i,j) as the average influence of node j on the correlations C(i,k), over all nodes k. The node dependencies define a dependency matrix D, whose (i,j) element is the influence of node j on node i. It is important to note that the dependency matrix is nonsymmetrical since the influence of node j on node i is not equal to the influence of node i on node j. The dependency matrix consists of mainly positive influences and few negative influences. In order to avoid cases when we sum over elements of different signs we choose to sum over positive influences only. *(Step 3)* Compute the *‘Influencing Degree’*- We define the influences of node j as the sum of the influence D(i,j) of j on all other nodes i. The higher this measure the more this node influenced all other connections in the network. *(Step 4)* Compute the *‘Influenced Degree’*- The dependency matrix is nonsymmetrical, therefore we can also sum all the influences (or dependencies) of all other nodes i in the network on node j - D(j,i). The higher the influenced degree measure the more this node was dependent or influenced by all the other nodes in the network. *(Step 5)* Creation of context related graph visualization – This graph captures both the *‘Influencing Degree’* (or *‘Influenced Degree’*) and the differences in dependencies between two different experimental conditions (or two different groups). All pairwise ROIs with dependency elements D that are significantly different at the p < 0.001 level are plotted as graph edges. This procedure allows for a simple graph visualization of the differences between the conditions across all subjects.

In addition, a visualization of the influence of nodes on a particular edge can be extracted from the analysis. All ROIs with correlation influence element d (see Equation 3 in the method section) that are significantly different at the q < 0.05 FDR corrected level are plotted as graph edges. This allows for a simple visualization of the influences on a specific edge in the network (i.e. upon the correlations between two specific ROIs) between conditions across all subjects. The D_EP_NA toolbox is available on request from the corresponding author.

### Simulations results

**Figure 2:**
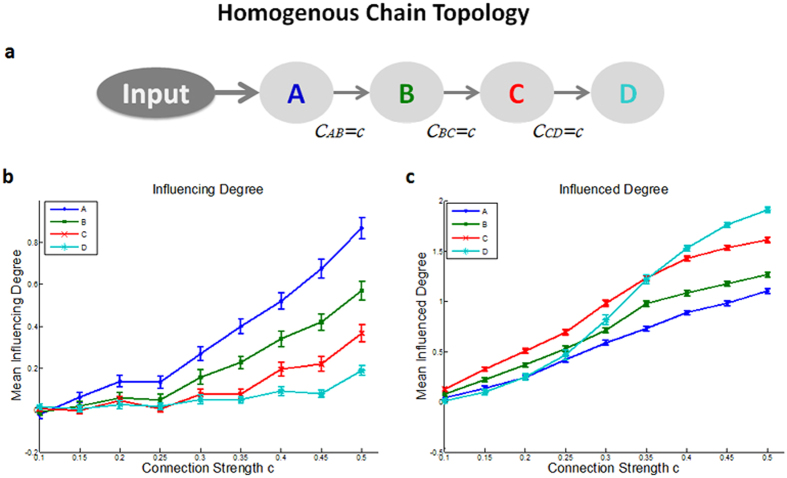
Simulated simple chain topology with homogenous connection strength results. **(a)** Illustration of the simple chain network topology. The arrows indicate the directed connection between the regions; the influence from one region to another, where c indicates the connection strength. Here we simulated a homologous network in which the connection strength between all regions is the same, while we adjusted it to range from a weak connection strength of 0.1 to a strong connection strength of 0.5. For each network connection strength the D_EP_NA was conducted on 200 randomized simulated BOLD signals (20 subjects X 10 trials). The D_EP_NA conducted on the simulated data results of ‘Influencing Degree’ **(b)** and ‘Influenced Degree’ **(c).** The D_EP_NA, as expected, correctly captured the network’s hierarchy of influence showing that the first region in the chain, which derives the simulated cascade of signals down the chain, has the highest ‘Influencing Degree’. Whereas, the last region in the chain, which only receives input and does not send output, has the lowest ‘Influencing Degree’. The ‘Influenced Degree’ exhibited an expected mirror effect, showing that the further downstream the node is in the network, the higher its ‘Influenced Degree’.

In order to test D_EP_NA robustness and its ability to correctly estimate the direction of the connection and influence hierarchy, we generated biophysical fMRI model simulations of several network scenarios. The simulation procedure was adopted from Smith *et al*.^20^, in which networks were used to simulate fMRI BOLD signals. The simulations were based upon the DCM fMRI forward model^21^. We examined four different simulations by adjusting different parameters;

(1) *Homogenous chain topology* - in order to examine the ability of the D_EP_NA to capture the true hierarchy of influence in the network, we simulated a simple chain topology network consisting of four regions, in which region A influences region B that in turn influences region C that in turn influences region D (see Fig. 2a). With all connection strengths in the network equal to one another. We then calculated the D_EP_NA measures for different connection strengths ranging from 0.1 to 0.5. The results revealed that the closer the region is to the input entrance point, the more influence it has on the network. As expected, the first region in the chain, which derives the simulated cascade of signals down the chain, has the highest *‘Influencing Degree’*. Whereas, the last region in the chain, which only receives input and does not send output, has the lowest *‘Influencing Degree’*. Therefore, the D_EP_NA correctly captured the network’s hierarchy of influence. This is true for all connection strengths ranging from 0.2 to 0.5 (see Fig. 2b). In addition, our results reveal that the stronger the connection strength (i.e. correlation coefficient), the higher the *‘Influencing Degree’*. However, although the last region in the chain, region D, should not be influencing upstream network regions, it was found to have a small degree of influence as connection strength was stronger.

For the *‘Influenced Degree’* we found an expected mirror effect to that of the *‘Influencing Degree’*, showing that the further downstream the node is in the network, the higher its *‘Influenced Degree’*. For example, while region B was influenced only by region A, region D was influenced by regions A, B and C, therefore its *‘Influenced Degree’* is expected to be higher. However, the *‘Influenced Degree’* results (demonstrated in Supplementary Figure S1), exhibited this result only for the three first regions in the chain, whereas, the forth region in the chain (i.e. region D) did not obtained the highest *‘Influenced Degree’* as would be expected. This is due to the signal’s decay over time, which is inherent in the simulation. Hence, the further downstream a region is along the path, the lower its signal to noise ratio (SNR); thus the D_EP_NA is unable to accurately capture its dependencies. Contrary to the *‘Influencing Degree’*, the *‘Influenced Degree’* was found to be much more sensitive to the SNR. Therefore, in order to achieve the expected correct results for the forth region in the chain we increased the size of the input signal, controlling for higher SNR. From here on we increased the SNR by a factor of 2 for all *‘Influenced Degree’* measures of all the simulations. As a result we were able to obtain the anticipated results, demonstrating that the further the region is down the path the higher its *‘Influenced Degree’*. This was true for all connections strengths, except for the last region in the chain (region D). Again, due to the SNR sensitivity, region D was correctly captured as having the highest *‘Influenced Degree’* only for connections strengths stronger than 0.3 (Fig. 2c). In addition, although region A should not be influenced by regions downstream, it was found to be influenced by some small degree that increased with connection strength.

**Figure 3:**
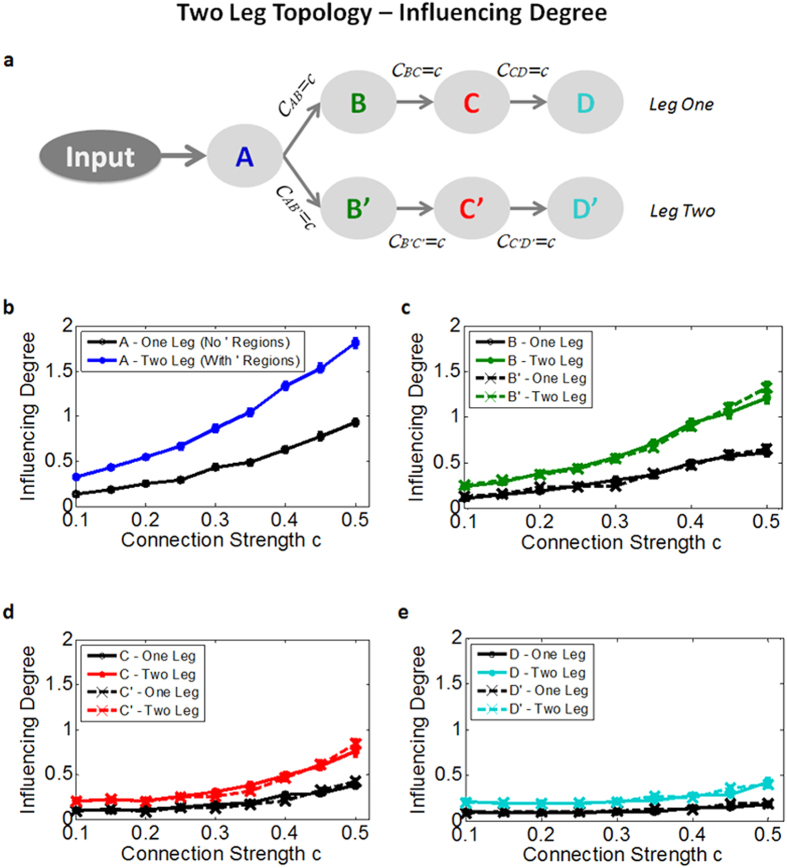
Simulated two-leg topology ‘Influencing Degree’ results. **(a)** Illustration of the two-leg network topology (as in Fig. 2). Here we simulated a network where a single region influences two separate regions creating two alternative paths. The D_EP_NA simulation results demonstrate the ‘Influencing Degree’ of the network regions in the two-leg versus one-leg scenario (simple chain topology as in Fig. 2), depicted in **(b**–**e)**. As expected, the influence of the first region (region A) increases significantly as it influences twice the regions compared to the one-leg scenario. In addition, although there is no direct connection between homologues regions (B and B’, C and C’ etc.), adding the second leg increases the ‘Influencing Degree’ of the regions downstream. Nevertheless, the network’s the influence hierarchy is preserved.

**Figure 4:**
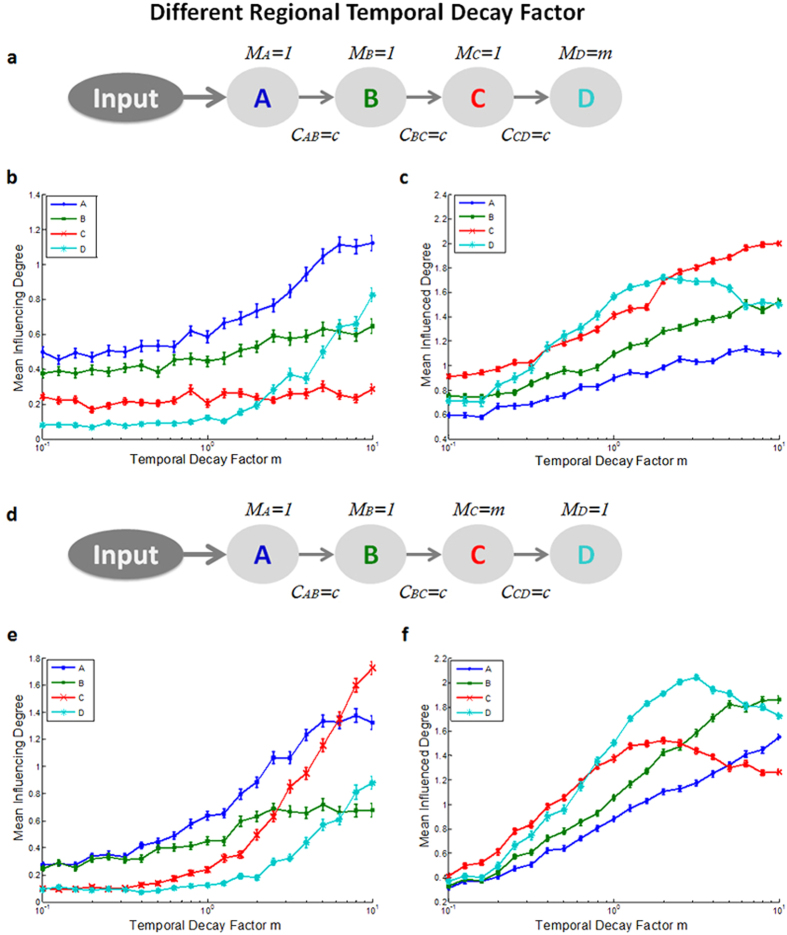
Adjusting regional temporal decay factor. Illustration of the simple chain network topology (as in Fig. 2), where m indicates the regions temporal decay time factor. **(a)** Here we simulated a network where a single region, region D, has a prolonged activation, representing the within region processing variability. **(b)** The D_EP_NA ‘Influencing Degree’ and **(c)** ‘Influenced Degree’ of the four regions, showing that increasing the temporal decay factor for the last region in the chain topology (region D), was found to significantly increase its ‘Influencing Degree’ and increased the general influence level in the network. **(d)** Increasing region C’s temporal decay factor, which is the third region in the chain, significantly increases its influence on the network, and above all, significantly increases the next region’s (region D) ‘Influencing Degree’ **(e)** and ‘Influenced Degree’ (**f**).

**Figure 5:**
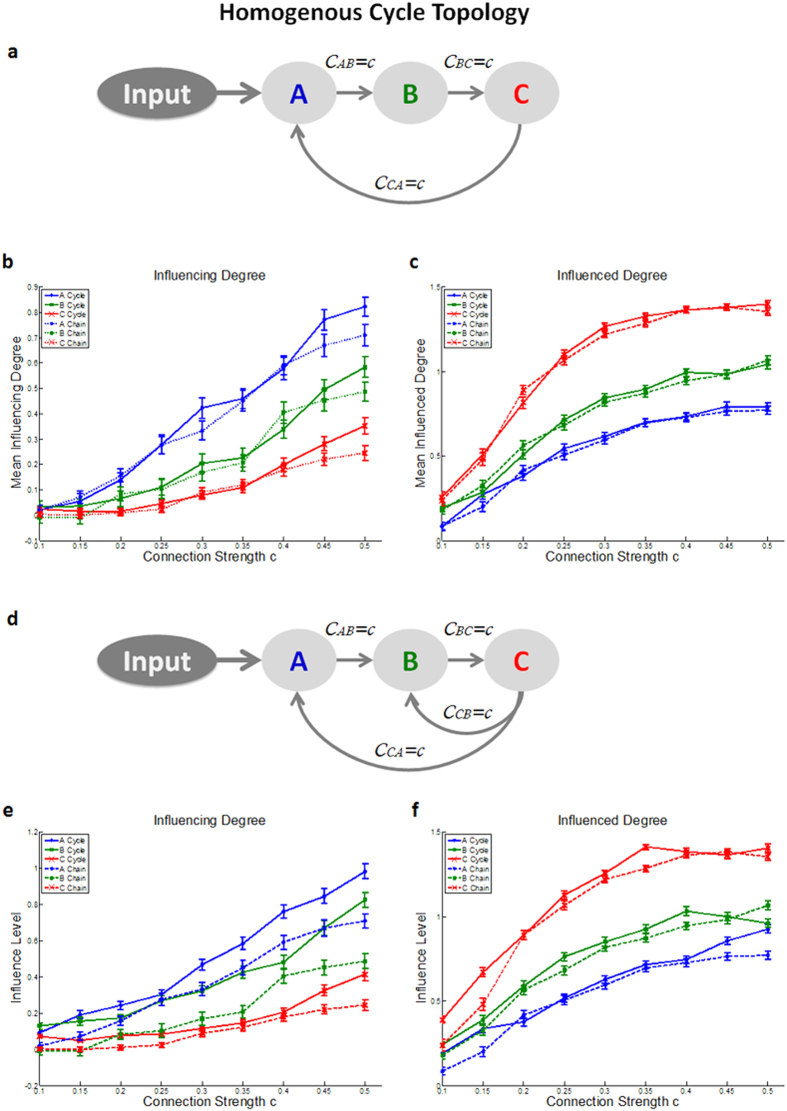
Simulated cycle topology with homogenous connection strength results. **(a)** Illustration of the simulated cycle network topology in which the last region, region C, influence back on the first region, region A. The D_EP_NA conducted on the simulated data results of **(b)** ‘Influencing Degree’ and **(c)** ‘Influenced Degree’. **(d)** Illustration of the simulated cycle network topology in which the last region, region C, influence back on both previous regions. The D_EP_NA conducted on the simulated data results of **(e)** ‘Influencing Degree’ and **(f)** ‘Influenced Degree’. As expected, and as in the simple chain topology network (Fig. 2), the closer the region is to the input entrance point, the more influence it has on the network. Moreover, closing a loop in the network, increases the *‘Influencing Degree’* and *‘Influenced Degree’* levels in general, whereas the overall direction of influence in the network is preserved.

(2) *Different mid connection strengths* - in order to examine the robustness of the D_EP_NA results we sought to test what happens if we strengthen or weaken only one connection in the chain. Therefore, we constructed the same simple chain topology, only this time adjusted only the middle connection strength between region B and region C (see Supplementary Figure S2), while fixing all other connection strengths to 0.4 (the mean connection strength according to Smith *et al*.^20^). Fixing the connection strength of the network and changing only the connection strength in the middle of the chain demonstrated the robustness of the D_EP_NA measure (see Supplementary Figure S2). The results show that strengthening this connection increases the *‘Influencing Degree’* of the first regions in the chain (i.e. A and B), whereas only a minor effect occurs for regions C and D downstream. The *‘Influenced Degree’* demonstrates the inverse effect, showing that strengthening this connection results in higher *‘Influenced Degree’* for the subsequent regions along the path (i.e. regions C and D).

(3) *Two leg topology* - in order to test a different network topology we added a second alternative pathway to the simple chain described above, where region A now influences two separate regions B and B’ that later cascade into two paths (legs) with no further connections between the paths after the separation (see Fig. 3a). The results revealed the expected increase in the influence of the first region (region A), as it influences twice the regions compared to the one-leg scenario (Fig. 3b). However, although there is no direct connection between homologues regions (B and B’, C and C’ etc.), adding the second leg increases the *‘Influencing Degree’* of regions downstream. This increase of influencing level of region B when two legs are present (Fig. 3c) is a property of the measure calculation and not of the topology of the network, since region B is not influencing B’. Nevertheless, the network’s influence hierarchy is preserved, demonstrating that the *‘Influencing Degree’* gradually decays down the network’s path. For the *‘Influenced Degree’* measure we obtain the opposite direction, showing that the last regions in the path (D and D’) have the highest *‘Influenced Degree’*, whereas the first region (region A) has the lowest values (see Supplementary Figure S3).

(4) *Different temporal decay factor* - under the assumption that a longer temporal decay of the averaged region’s BOLD signal reflects longer local field potential (LFPs) activity^22^,^23^,we hypothesize that these prolonged BOLD activations (or sustained neuronal activation) will increase the impact of this region on the network and thus increase its *‘Influencing Degree’*. We thus conducted the simulation again on the simple chain topology (all connections were set to 0.4, which is the mean connection strength according to Smith *et al*.^20^), only this time we adjusted the region temporal decay of a specific region by factor m, ranging from 0.1 to 10 (see Fig. 4a). Increasing the temporal decay factor for the last region in the chain topology (region D) was found to significantly increase its *‘Influencing Degree’* and to increase the general influence level in the network (Fig. 4b). In addition, when increasing the temporal decay of the third region in the chain, region C, again, its *‘Influencing Degree’* measure significantly increased. Above all, region D’s *‘Influencing Degree’* increased as well (Fig. 4e).

(5) *Homogenous cycle topology* - in order to examine the D_EP_NA performance on a more realistic brain network topology, we simulated cycle topologies networks (see Fig. 5a,d). With all connection strengths in the network equal to one another. We then calculated the D_EP_NA measures for different connection strengths ranging from 0.1 to 0.5. The results revealed that as expected, and as in the simple chain topology network, the closer the region is to the input entrance point, the more influence it has on the network. Therefore, the D_EP_NA correctly captured the network’s hierarchy of influence. In addition, as expected, adding a connection of influence between the last region, region C, and the first region, region A, increased the *‘Influencing Degree’* level of region C and consequently the *‘Influencing Degree’* of the now downstream regions A and B (Fig. 5b). The *‘Influenced Degree’* measure however, did not seem to change considerably (Fig. 5c). Adding a second loop by adding a connection of influence between region C and region B (Fig. 5d), showed again as expected that D_EP_NA correctly captured the network’s hierarchy of influence, and also showed an increase in the *‘Influencing Degree’* level of region C and consequently the *‘Influencing Degree’* of regions A and B (Fig. 5e). The *‘Influenced Degree’* showed a slight increase in the level of dependency of all three regions (Fig. 5f).

### Empirical fMRI data results

Data for this study consisted of scans from 100 subjects provided in the Q2 data release of the HCP^24^,^25^. We used data from two separate imaging conditions: motor and working memory.

### Motor task results

This task was adapted from Buckner and colleagues^26^. Participants were presented with visual cues instructing them to either tap their left or right fingers, or squeeze their left or right toes. The motor network included a set of regions that are consistently activated during hand or foot movement^27^,^28^; the bilateral precentral gyrus, supplementary motor area (SMA), thalamus and cerebellum. Overall the motor network consisted of 8 regions of interest (ROIs). The principal eigenvariate (time series) was extracted for each ROI mask image and each subject and then averaged across all left hand/foot movement blocks and right hand/foot movement blocks separately.

**Figure 6:**
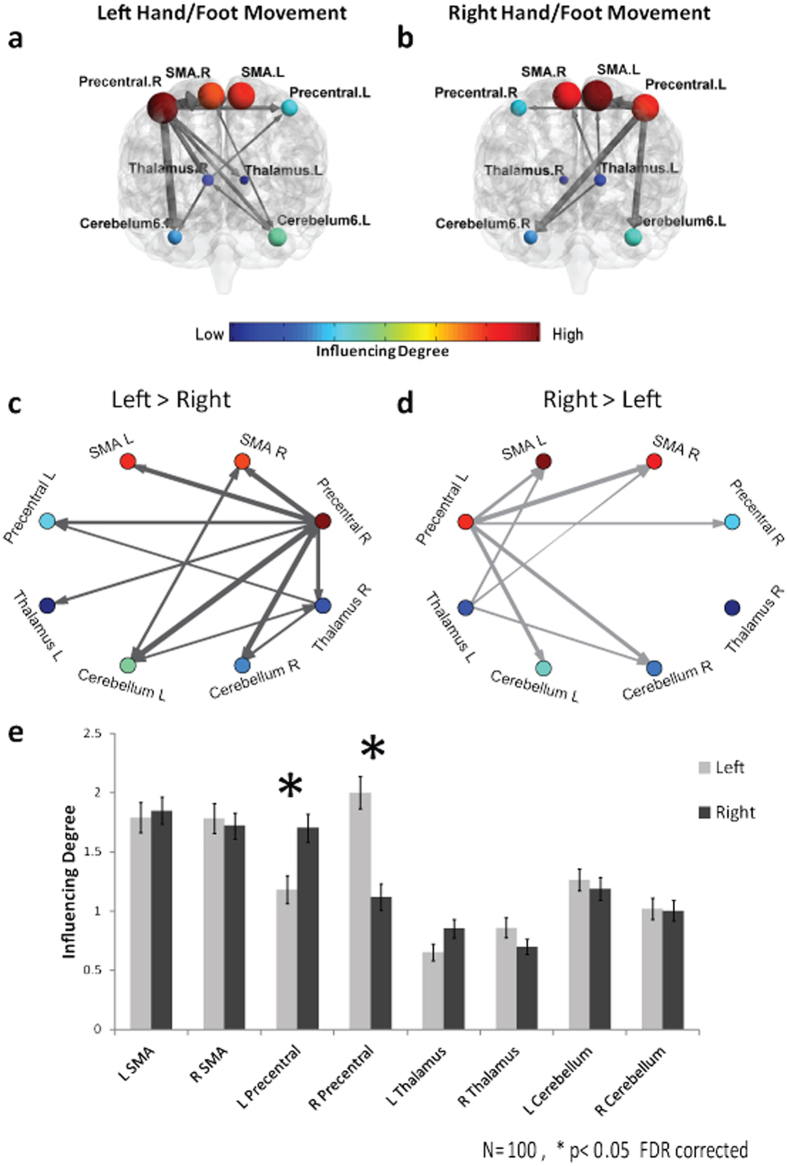
D_EP_NA conducted on a motor task. The ‘Influencing Degree’ **(a)** and ‘Influenced Degree’ **(b)** network illustrations. Each region is color coded according to his averaged ‘Influencing Degree’ or ‘Influenced Degree’ over all subjects. All pairwise ROIs with connections that were significant at the p < 0.05 level are plotted as edges Graph visualization of the motor network ‘Influencing Degree’ **(c)** and ‘Influenced Degree’ **(d)** (Different visualization of the same results as in A and B respectively). **(e)** The nodes’ ‘Influencing Degree’ averaged over all 100 subjects. Left hand/foot movement condition involved increased influence of right precentral gyrus compared to right hand/foot movement condition, and vice versa, the right hand/foot movement condition involved increased ‘Influencing Degree’ of left precentral gyrus.

Applying the D_EP_NA to left hand/foot movement blocks revealed that the contralateral right precentral gyrus on average had the highest influence on the network (average *‘Influencing Degree’* = 1.6), whereas analysis of the left hand/foot movement blocks revealed that the contralateral left SMA had the highest average influence on the network (average *‘Influencing Degree’* = 1.48) (Fig. 6b). Comparison of the influence degrees of each ROI between the two conditions revealed that the left precentral gyrus in the contralateral right hand/foot movement condition had a greater influence on the network compared to the ipsilateral condition (t = −4.59, p = 1.28e-05 FDR corrected), and the right precentral gyrus in the contralateral left hand/foot movement condition had a greater influence on the network compared to the ipsilateral condition (t = 5.84, p = 6.67e-08 FDR corrected) (see Fig. 6c and Table 1). As for the *‘Influenced Degree’* measure, none of the regions were found to be more influenced by all other regions in the network in the left hand/foot condition compared to the right hand/foot condition.

In addition, visualization of the influences of nodes on a particular edge (i.e. upon the correlations between two specific ROIs) is presented in the supplementary information Figure S4. These results demonstrate for example that the correlation between the right and left cerebellum is significantly influenced by the right precentral gyrus in the left hand/leg movement condition, and by the left precentral gyrus in the right hand/foot movement condition.

### Working memory task results

For the working memory task we used the N-back paradigm in which subjects are presented with a sequence of visual stimuli (pictures of places, tools, faces and body parts), and asked them to indicate if the current stimulus matches one n steps earlier in the sequence. Participants were presented with blocks of trials that consisted of either 0-back or 2-back loads. Working memory network was defined according to a meta-analysis of the N-back paradigm by Owen *et al*.^29^. The network included a total of 12 ROIs: the bilateral premotor, right SMA, bilateral dorsolateral prefrontal cortex (DLPFC), bilateral ventrolateral prefrontal cortex (VLPFC), left frontal pole, medial posterior parietal, bilateral inferior parietal lobule (IPL) and medial cerebellum. The principal eigenvariate (time series) was extracted for each ROI mask image and each subject. These signals were then averaged across all of the 2-back blocks and across all of the 0-back blocks separately.

**Figure 7:**
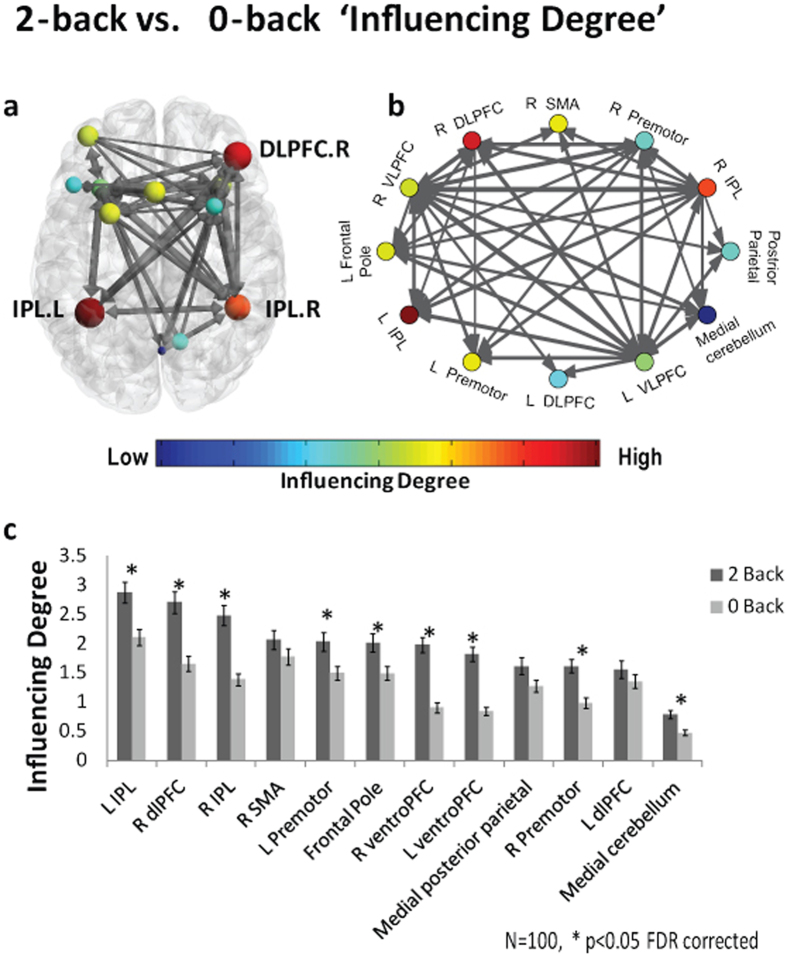
D_EP_NA conducted on a working memory N-back task- ‘Influencing Degree’ results. The working memory network illustration on an axial view **(a)** and graph visualization **(b)**. Each region is color coded according to his ‘Influencing Degree’ during 2-back condition (see colored scale). All pair-wise ROIs with connections that were significant at the p < 0.001 level are plotted as edges. **(c)** The nodes’ ‘Influencing Degree’ averaged over all 100 subjects. The right DLPFC, medial cerebellum, frontal pole, bilateral VLPFC, premotor and IPL showed a significant increase in their ‘Influencing Degree’ in the 2-back condition compared to 0-back condition (p < 0.05 FDR corrected).

**Figure 8:**
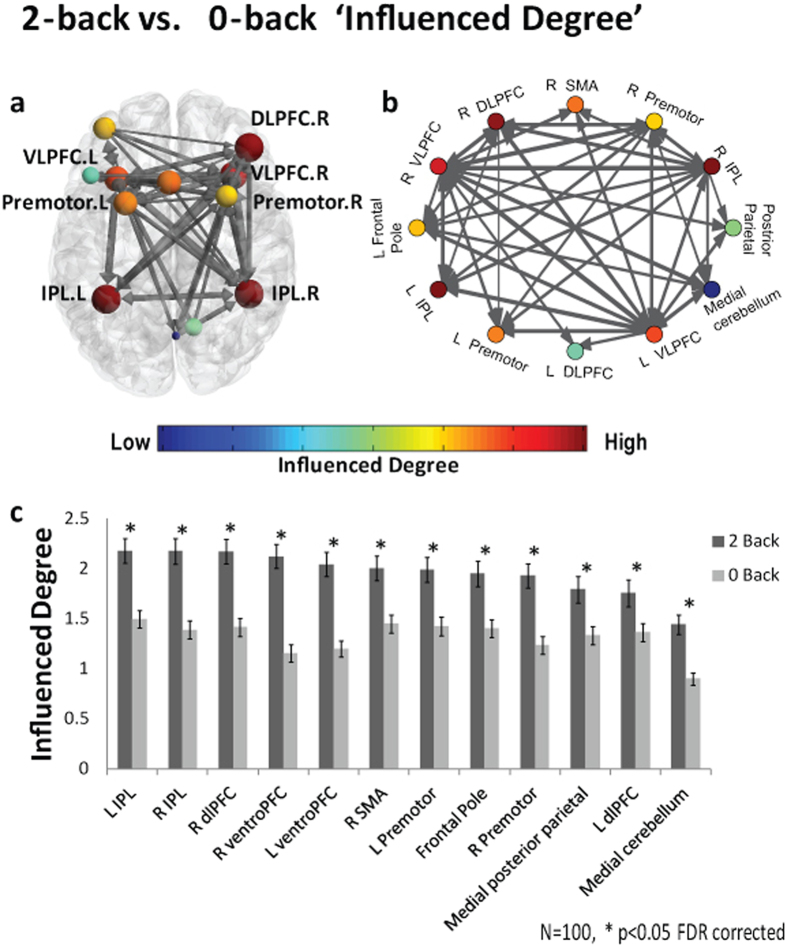
D_EP_NA conducted on a working memory N-back task- ‘Influenced Degree’ results. The working memory network illustration on an axial view **(a)** and graph visualization **(b)**. Each region is color coded according to its ‘Influenced Degree’ during 2-back condition. All pair-wise ROIs with connections, significant at the p < 0.001 level, are plotted as edges. **(c)** The nodes’ ‘Influenced Degree’ averaged over all 100 subjects. All of the network regions showed a significant increase in their ‘Influencing Degree’ in the 2-back condition compared to 0-back condition (p < 0.05 FDR corrected).

The analysis revealed that the performance of the 2-back condition involved increased influence of almost all working memory network regions compared to the 0-back condition. The ‘*Influencing Degree’* and *‘Influenced Degree’* are shown in Figs 7 and 8 respectively. The *‘Influencing Degree’* was higher during the 2-back condition than during the 0-back epoch in right DLPFC (t = 4.8, p = 5.64e-06), right VLPFC (t = 6.59, p = 2.11e-09), left VLPFC (t = 7.1, p = 1.9e-10), right premotor (t = 4.95, p = 3.05e-06), left premotor (t = 2.90, p = 0.0046), right IPL (t = 5.94, p = 4.28e-08), left IPL (t = 3.79, p = 0.00026), frontal pole (t = 2.98, p = 0.0036) and medial cerebellum (t = 3.89, p = 0.00018) all FDR corrected (see Table 2).

All of the network regions showed a significant increase in *‘Influenced Degree’* (p < 0.05 FDR corrected).

## Discussion

The current study introduces a new approach for the analysis of the dynamics of task related functional networks, as depicted by fMRI, entitled Dependency Network Analysis (D_EP_NA). Using partial correlations, D_EP_NA calculates how much the correlation between two nodes is influenced by a third node. Formally, partial correlation is a measure of the strength and direction of a linear relationship between two continuous variables whilst controlling for the effect of a third continuous variable. Thus, it obtains the clean correlation between two nodes X and Y when regressing out the influence of a third node Z. In order to quantify how much node Z influenced the correlation between nodes X and Y, we calculate the ‘correlation influence’ which is the correlation between X and Y minus the partial correlation between them given Z. That leaves us with just the influence that Z had over the correlation between nodes X and Y. Therefore, the ‘correlation influence’ measure is not a measure of correlation (i.e. co-linearity between two signals), rather a quantity of the effect a third node signal had over the correlation. Meaning, this quantity is large only when a significant fraction of the correlation between node X and node Y can be explained in terms of Z (for more details see Kenett *et al*.^17^). Then, the dependency matrix element is just the sum on all influences of node Z on the correlations of node X with all other nodes in the network. We thus obtain a dependency matrix which is a directed matrix as it is asymmetric, because the influence of node Z on node X is not the same as the influence of node X on node Z. Finally, we quantify for each node with an *‘Influencing Degree’*, defined as the sum of influences of the node on all other nodes in the network, and *‘Influenced Degree’* as the sum of influences of all other nodes on it.

Our results demonstrate the ability of D_EP_NA to capture the true direction of the influence using a biophysical data simulation. Importantly, we show that the closer the region is to the entering point of information, or the longer the region processes the information, the higher it’s influence on the entire network. Using empirical fMRI data from two tasks derived from 100 participants we showed the ability of the D_EP_NA to compare features from different graphs, thus demonstrating its ability to explore functional influence hierarchies across various experimental conditions.

### Depicting the topological characteristics of D_EP_NA via simulated data

Conducting the D_EP_NA on a biophysical simulated dataset we demonstrated that the D_EP_NA was successful in capturing the network’s hierarchy of influence as anticipated. More specifically, we showed that the region generating the cascading influence exhibited the highest *‘Influencing Degree’*; whereas the region that merely received input and did not send out any output had the lowest *‘Influencing Degree’*. Therefore, the region’s *‘Influencing Degree’* measure indeed reflects its correct relative place in the influence hierarchy; thus regions with a high *‘Influencing Degree’* are more likely to generate the cognitive process.

In addition, we demonstrated that the stronger the connection between nodes, the higher the overall influence level of the network. In order to demonstrate D_EP_NA robustness, we also showed that the overall influence level hierarchy was found to be insusceptible to changes in connection strength or changes in the strength of only one connection in the chain (see Supplementary Figure S2). Therefore, the D_EP_NA successfully captures the correct influence hierarchy, even when a single connection between two regions is relatively weak.

For the *‘Influenced Degree’* we found an expected mirror effect, showing that the further downstream the node is in the network, the higher its *‘Influenced Degree’*. In a specific node, the *‘Influenced Degree’* is the inverse of the *‘Influencing Degree’* in the sense that its direction of change is opposite as a function of the nodes’ distance from input (Fig. 2). Nevertheless, both measures in a selected node can be relatively high or low. It is important to note that as opposed to the *‘Influencing Degree’*, the *‘Influenced Degree’* was found to be very sensitive to the SNR of the input signal; the D_EP_NA might miss a later activated node in a simple chain network, therefore this measure should be interpreted with some caution (see Supplementary Figure S1). However, importanly the D_EP_NA is successful in capturing regions that are simultaneously influenced by many other regions.

Following the assumption that the BOLD fMRI signal reflects neural activity of LFPs^22^,^23^, we hypothesized that a longer temporal decay of the BOLD signal reflects sustained activity. We found that a regional increase in the temporal decay of the signal significantly increased its *‘Influencing Degree’*, and further increased the *‘Influencing Degree’* of regions further downstream the path. Therefore, another crucial outcome of the simulation is that D_EP_NA also takes into account the regional temporal decay of the signal, assigning additional influence to regions that may involve higher processing levels.

Conducting the simulation on a different network topology of a single region cascading into two separate chains of regions (Fig. 3a), demonstrated that D_EP_NA measures are highly influenced by correlations and not only by the causality of time. Therefore, regions that have no direct connection between them but exhibit similar signal patterns will be influenced by one another; however, the network’s influence hierarchy is preserved. This is a crucial point indicating that D_EP_NA measures do not infer causal influence in a true sense, rather infer the network’ hierarchy of influence based on correlational influences.

Additionally, although the last region in the chain is not thought to influence the upstream network regions, and the first region in the chain thought not to be influenced by downstream regions, they were however found to have a small degree of such an influence (Fig. 2). This residual influence effect might results from the fact that correlation values between regions always have a residual non-zero value even if the expected correlation is zero.

Finally, various studies have shown that anatomical brain networks exhibit a modular structure consisting of high clustering coefficients and closed loops^30^,^31^. Therefore, in order to examine the D_EP_NA performance on a more realistic brain network topology, we conducted simulations of different cycle topologies. As anticipated, these simulations revealed that, as in the simple chain topology network, the closer the region is to the input entrance point the more influence it has on the network. Moreover, closing a loop in the network increases the *‘Influencing Degree’* and *‘Influenced Degree’* levels in general, while the overall direction of influence in the network is preserved.

### Revealing the functional validity of D_EP_NA via empirical data

We further applied D_EP_NA to two empirical fMRI datasets using time courses of pre-selected brain networks. Relying on existing prior knowledge regarding the functional properties of the brain regions examined here, we show that D_EP_NA is an effective method for quantifying the correlational influence between nodes in a network. As expected, examining fMRI data obtained during a lateralized motor task revealed higher influence of the contralateral, rather than the ipsilateral, precentral gyrus for motor movement. This finding is in line with the well known critical role of the contralateral precentral gyrus region in motor execution (see review by Beaul *et al*.^32^). In addition, the bilateral SMA region showed no difference between conditions, in accordance to the less strict lateralization of this medial region in the control of movement^33^. Furthermore, the bilateral SMA showed the highest averaged *‘Influencing Degree’* values, which is in line with the known role of the SMA in generation and initiation of movement sequences^32^,^34^,^35^. As was shown in different electrophysiological and fMRI studies, the SMA is known to be activated up to 2 seconds prior to movement initiation, preceding other motor regions^36^,^37^,^38^. Therefore, its high *‘Influencing degree’*, regardless of lateralization, is highly expected. This result demonstrates the strength of the D_EP_NA in identifying the sequential temporal contribution of network regions in real fMRI data; in spite of its poor temporal resolution, thus confirming its ability to infer causal relations within a network using fMRI data.

In the second data set we examined the effect of a working memory task using the N-back paradigm. We found that several ROIs including the right DLPFC, right premotor, bilateral VLPFC and bilateral IPL had greater influence on the network (i.e. *‘Influencing Degree’*) during the 2-back condition compared to 0-back. Specifically, the bilateral IPL and right DLPFC showed the highest *‘Influencing Degree’* values. Cohen *et al*.^39^ have shown that the right DLPFC and IPL regions exhibit a sustained activation signal pattern (as opposed to transient signal), suggesting that these regions play a key role in the active maintenance of working memory. It is possible that the observed increase in the *‘Influencing Degree’* of these regions when the working memory load is higher, could be attributed to their sustained activation quality, an effect that was shown in the simulated data results (Fig. 4). Importantly, these results demonstrate the ability of D_EP_NA to extract the influence level hierarchy, assigning higher *‘Influencing Degree’* to regions that play a significant role in the cognitive task and involve higher levels of processing.

Furthermore, the right DLPFC, right premotor, frontal pole and bilateral IPL have been previously suggested to play a role in nonverbal identification N-back tasks^29^, such as was performed in this dataset. Therefore, we used a working memory network constructed from a meta-analysis of all N-Back experiment types. Nevertheless, the *‘Influencing Degree’* marked regions that are more specific to the nonverbal identification N-Back task type. Therefore, D_EP_NA can successfully find regions that play a substantial role in the cognitive task and change their influence level significantly between two conditions.

Regarding the *‘Influenced Degree’* measure, all of the network regions showed a significant increase in the 2-back compared to 0-back, indicating that all of these regions were more influenced by each other as the working memory load increased. This result is interesting, demonstrating that under higher cognitive loads regions in the network are more influenced by each other, thus the network is more interconnected. Furthermore, this result highlights the difference in the changing relations of nodes in a network, as influencing or being influenced, under specific conditions. Here for example, we found that under high vs. low cognitive load few regions exhibited higher *‘Influencing Degree’*, while the entire network regions were found to be more influenced.

In line with the known role of the VLPFC as the end point of the ventral pathway, which processes information about the stimuli’s characteristics^40^ and plays an important role in behavioral inhibition, for example as in go-no go tasks^41^,^42^, we found that the bilateral VLPFC exhibited the highest *‘Influenced Degree’* in the network. We hypothesize that the *‘Influenced Degree’* measure marked the VLPFC as the end point of the cognitive process taking place in the N-back paradigm. Therefore, the VLPFC gathers information from many other regions in the network in order to execute a decision. This result demonstrates the ability of the D_EP_NA to identify such hubs in the network that receive a lot of information (input) as shown in real fMRI data.

Altogether the empirical fMRI dataset findings demonstrate the ability of D_EP_NA to allocate important influential regions in experimentally-induced coupling within a relevant functional network.

### D_EP_NA strengths and limitations

D_EP_NA offers a new way of constructing a context specific directed graph that is principally based on partial correlations. In this regard, D_EP_NA does not rely on the temporal resolution of the analyzed signal, which is an advantage given the poor temporal resolution of fMRI.

D_EP_NA probes the hierarchies and the influence within the network. In addition, D_EP_NA reveals crucial information that is not provided by standard analysis of BOLD activation; information regarding connectivity in the network and the influence hierarchies. Figure S5 in the supplementary information presents the different network hierarchies outlined by BOLD activation magnitude and by D_EP_NA. Moreover, D_EP_NA allows for the study of the influences on specific nodes (e.g. Fig. 6c,d) and also on explicit edges (i.e. connections in the network) (Figure S4).

Most fMRI studies to date have been based on the pair-wise correlation matrix resulting in undirected graphs of brain networks^13^. Few studies have applied Granger causality on resting state fMRI data in order to construct a directed graph^14^,^15^, with these studies constrained by the temporal resolution required by the Granger methodology. D_EP_NA offers a new way of constructing a directed graph that is based on correlations. This is of great importance in the field of neuroimaging such as fMRI in which the only information regarding communication between regions is based on correlations.

As opposed to other functional connectivity methods such as PPI, D_EP_NA allows for an integrative measure of coupling between different nodes in the network (as opposed to one network node at a time), thus the study of context dependent brain states, as well as revealing the functional hierarchy within and between networks. Compared to data-driven functional connectivity methods such as ICA and PCA, D_EP_NA addresses the dynamic relationships among nodes of a given network or between networks which can be used to underlie functional hierarchies^5^. Such hierarchies can then better characterize the functional specificity of a network^6^.

D_EP_NA does not require any specific a-priori assumption of direction of influence models as opposed to other model-driven network methods, such as the DCM. Nevertheless, the main limitation of D_EP_NA is that it does not infer causal influence in the true sense, since “data cannot cause data; data are caused by underlying brain states”, as stated by Friston *et al*.^43^. Therefore, this method is somewhere between functional connectivity and effective connectivity and can be used to make inferences about the influence hierarchy of a network. We therefore suggest that D_EP_NA may serve as a pre-test for other causality methods, such as the DCM, to delineate the a-priori model of critical nodes and correlational influences in a specific network.

While this method can be easily implemented in a data-driven manner on whole-brain data, such an approach also entails important limitations. It might be sub-optimal in cases of modular processing, in which a specific psychophysiological function is related to a specific network rather than to whole-brain activity. In this case, key features of the influence within the functional network might be masked when accounting for a large set of irrelevant influences. Therefore, we speculate that conducting D_EP_NA on whole brain interactions will mainly depict critical regions that show a general overall non-specific effect.

Most centrality measures, such as node degree, betweenness and closeness centrality^44^, require a certain threshold on the complete graph in order to create an adjacency matrix. Thus this arbitrary choice of threshold can result in completely different findings of the network hubs. Other centrality measures such as eigencentrality^44^ can use the weighted matrix of the fully connected graph (i.e. all nodes are connected to all nodes) in order to calculate node centrality. However, these measures are not appropriate for correlation networks which contain negative edges, unless the edge weight sign is ignored.

Lastly, the dependency matrix consists of mainly positive influences and few negative influences which could be considered as suppressors^45^,^46^. Therefore, if we sum over elements of different signs a node’s influence degree could be nearly zero but may still exert influence over, or be influenced by, specific nodes in the network. In order to deal with this issue we defined the D_EP_NA to sum over positive influences only. Conversely, one can also argue that suppressors might also be influential, then we suggest to sum over the magnitude of the influences (i.e. absolute values). Nevertheless, it is important to note that if these negative influences are smaller than the sample’s standard deviation it is reasonable to assume that they do not indicate suppression, rather they reflect the variance of the sampling. Future studies should further investigate negative influences and their interpretation.

In addition, we suggest that if one has a specific assumption regarding a particular module in the brain network, D_EP_NA can assign different influence degrees of the node regarding each different module.

## Conclusions

D_EP_NA offers a new computational way of looking at the direction of influence among nodes within a given brain network during task performance. By pointing to functional influential hierarchy within a network D_EP_NA could ultimately facilitate in deciphering important network hubs that underlie specific cognitive processes. This targeting is of clear importance from both a theoretical and clinical point of view. From a clinical perspective for example, D_EP_NA could help identify the most effective target regions for neuro-modulation techniques such as transcranial magnetic stimulation (TMS), transcranial direct current stimulation (tDCS) and neurofeedback.

## Methods

### Dependency network analysis (D_EP_NA)

#### The ROI-ROI correlations

The Pearson correlation of BOLD signals in different brain regions is widely used to obtain functional networks from fMRI signals^28^,^47^. Moreover, it has been shown that linear correlation captures most of the interaction, and is a very good tool to study functional connectivity graphs^48^,^49^.

The ROI-ROI correlations was calculated by Pearson’s formula^50^:


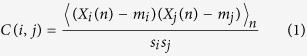


where *X*_*i*_ (*n*) and *X*_*j*_ (*n*) are the signal activity of ROIs *i* and *j* of subject *n, m* stands for average, and *s*_*i*_ and *s*_*j*_ are the standard deviation (STD) of the dynamics profiles of ROIs *i* and *j*. Note that the ROI-ROI correlations for all pairs of ROIs define a symmetric correlation matrix whose (*i, j*) element is the correlation between ROIs *i* and *j*.

### Partial correlations

Next, we used the resulting ROI correlations to compute partial correlations^51^ (Fig. 1a). The first order partial correlation coefficient is a statistical measure indicating how a third variable affects the correlation between two other variables^52^. The partial correlation between nodes i and k with respect to a third node j – *PC (i, k*|*j*) is defined as:


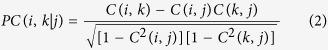


where *C (i, j*), *C (i, k*) and *C (k, j*) are the ROI-ROI correlations.

### The correlation influence and correlation dependency

The relative effect of the correlations *C (i, j*) and *C (k, j*) of node *j* on the correlation *C (i, k*)^17^, is given by:


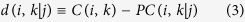


This approach avoids the trivial case where node *j* appears to strongly affect the correlation *C (i, k*), mainly because *C (i, j*), *C (i, k*) and *C (k, j*) all have small values. We note that this quantity can be viewed either as the correlation dependency of *C (i, k*)on node *j*, or as the correlation influence of node *j* on the correlation *C (i, k*)^17^ (Fig. 1a). Also, this quantity is large only when a significant fraction of the correlation between nodes i and k can be explained in terms of node j. There might be cases of negative influences which could be considered as suppressors^45^,^46^. In order to avoid cases where we sum over positive and negative influences, we chose to reset all negative values to zero. However, one can also choose to look at the absolute values of the influences.

### ROI activity dependencies

We then define the total influence of node j on node i, or the dependency *D (i, j*) of node i on node j to be:


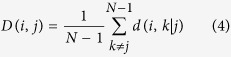


As defined, *D (i, j*) is a measure of the average influence of node j on the correlations *C (i, j*), over all nodes k. N is the number of nodes in the network. The node activity dependencies define a dependency matrix D whose (*i, j*) element is the influence of node j on node i. It is important to note that while the correlation matrix C is a symmetric matrix, the dependency matrix D is nonsymmetrical – *D (i, j*) ≠ *D (j, i*), since the influence of node j on node i is not equal to the influence of node i on node j (Fig. 1b).

### The ROI ‘Influencing Degree’ and ‘Influenced Degree’

Next we sorted the nodes according to the system level influence of each node on the correlations between all other node pairs (Fig. 1c). The system level *‘Influencing Degree’* of node j is simply defined as the sum of the influence of node j on all other nodes i, that is:


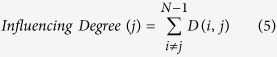


The higher the *‘Influencing Degree’* measure the more this node influences all other connections in the network. The above definition is for the outgoing influence; however a similar definition can be used to define the incoming influence of the combined dependencies.

The influence of the network on node j is defined as the sum of the influences (or dependencies) of all other nodes i in the network on node j, that is:


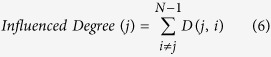


The higher the *‘Influenced Degree’* measure the more this node was dependent or influenced by all the other nodes in the network (Fig. 1d).

The D_EP_NA toolbox is available on request from the corresponding author.

### Simulations

The simulation procedure was adopted from Smith *et al*.^20^. The description of the simulation is detailed as follows:

First, we calculated the signal propagation in time, given by:


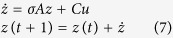


where z is the neural activity time series and, σ is the neural lag, set to 50 ms. The vector *u* indicates the external input according to the simulated experiment paradigm, where *u* equals one when the stimuli is presented, and zero when resting. C is the weights matrix controlling how the external input is fed into the system. We set such


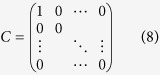


that the external input is always fed only to the first region. Off-diagonal elements in matrix A determines the network connection strength between regions, where the diagonal elements model within region temporal decay. The effect of the within-node dynamics (exponential temporal decay) creates a lag between the input and output of every node. The matrix A was different for each simulation according to the connection strength or temporal decay.

The BOLD signal is then given by:


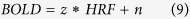


where HRF is the hemo-dynamic response function, * is the convolution operation, and n is the white Gaussian random noise (thermal noise) with a mean of 0.1 and standard deviation of 0.9. The BOLD signal was then down sampled to the experiment time resolution (TR = 2.2 seconds).

The simulation paradigm was based on a typical fMRI block design experiment consisting of 20 subjects. The paradigm consisted of 10 blocks; each block lasted 22 seconds (10 trials). We then calculated the neural network model to receive a simulated BOLD signal for each subject and each trial. Next we used the acquired simulated BOLD signal to calculate the *‘Influencing Degree’* and *‘Influenced Degree’* scores per trial per subject. Therefore, we obtained 200 scores (10 trials X 20 subjects) and averaged across trials and subjects to receive the final scores of *‘Influencing Degree’* and *‘Influenced Degree’* along with their standard error of the mean.

We examined four different simulations by adjusting different parameters in matrix A;

(1) *Homogenous chain topology* - a simple chain topology network consisting of four regions, with all connection strengths in the network equal to one another (see Fig. 2a). We then calculated the D_EP_NA measures for different connection strengths ranging from 0.1 to 0.5. Diagonal elements were set to −1 according to Smith *et al*.^20^.

(2) *Different mid connection strengths* - a simple chain topology, only this time adjusted only the middle connection strength (see Supplementary Figure S1), while fixing all other connection strengths to 0.4 (the mean connection strength according to Smith *et al*.^20^), and diagonal elements set to −1.

(3) *Two leg topology* - adding a second alternative pathway to the simple chain described above, where the first region influences two separate regions that later cascade into two paths (legs) with no further connections between the paths after the separation (see Fig. 3a). Diagonal elements were set to −1 according to Smith *et al*.^20^.

(4) *Different temporal decay factor* – a simple chain topology (all connections were set to 0.4), only this time we adjusted the region temporal decay of a specific region by dividing its diagonal element in the matrix by factor m, ranging from 0.1 to 10 (see Fig. 4a).

### Empirical fMRI Datasets

The datasets used in this work came from the Human Connectome Project^24^,^25^, derived from 100 healthy participants. All experiments were performed in accordance with the relevant guidelines and regulations of the Human Connectome Project, WU-Minn Consortium (Principal Investigators: David Van Essen and Kamil Ugurbil; 1U54MH091657) funded by the 16 NIH Institutes and Centers that support the NIH Blueprint for Neuroscience Research; and by the McDonnell Center for Systems Neuroscience at Washington University, which approved all the experimental protocol and procedures. Written informed consent was obtained for every participant in the study.

All scans were obtained by a Siemens Skyra 3T scanner with a 32-channel head coil located at Washington University in St. Louis. The acquisition parameters of the task fMRI (tfMRI) data were: 90 × 104 matrix, 220 mm FOV, 72 slices, TR = 0.72 s, TE = 33.1 ms, flip angle = 52°, BW = 2290 Hz/Px, in-plane FOV = 208 × 180 mm, 2.0 mm isotropic voxels.

The preprocessing pipeline included motion correction, spatial smoothing, temporal pre-whitening, slice time correction, global drift removal. All of these steps are implemented by FSL FEAT^53^,^54^. For more detailed data acquisition and preprocessing see^24^,^25^.

### Motor task

This task was adapted from Buckner and colleagues^26^. Participants were presented with visual cues instructing them to either tap their left or right fingers, or squeeze their left or right toes. Each block involved a different movement type and lasted 12 seconds (10 movements), preceded by a 3 seconds cue. The task included 8 blocks (4 hand movement blocks, 2 right and 2 left, and similarly 4 foot movement blocks).

The motor network included a set of regions that are consistently activated during hand or foot movement^27^,^28^. Overall the motor network consisted of 8 regions of interest (ROIs). These ROIs were defined according to the Wake Forest University (WFU) PickAtlas^55^. The principal eigenvariates (time series) were extracted for each ROI mask image and each subject using SPM8 (Wellcome Department of Cognitive Neurology, London, UK, http://www.fil.ion.ucl.ac.uk/spm/). These signals were then averaged across all left hand/foot movement blocks and right hand/foot movement blocks separately.

### Working memory task

The N-back paradigm was used as a working memory task, in which participants are presented with a sequence of visual stimuli (pictures of places, tools, faces and body parts), and asked to indicate if the current stimulus matches one n steps earlier in the sequence. Participants were presented with blocks of trials that consisted of either 0-back or 2-back loads. The dataset included 4 2-back blocks and 4 0-back blocks. A 2.5 second cue indicated the task type (and target for 0-back) at the start of the block. Overall the task contained 8 task blocks of 25 seconds (10 trials of 2.5 seconds each) and 4 fixation blocks (15 seconds). On each trial, the visual stimulus was presented for 2 seconds, followed by a 500 ms inter-task interval (ITI).

Working memory network was defined according to a meta-analysis of the N-back paradigm by Owen *et al*.^29^. The network included a total of 12 ROIs. The coordinates reported in Owen *et al*.^29^ were converted from Talariech to MNI space using tal2icbm^56^,^57^. For each ROI, we created spherical masks (radius = 8 mm, volume = 2376 mm^3^) centered on the peak x, y, z MNI coordinates using SPM8 (Wellcome Department of Cognitive Neurology, London, UK, http://www.fil.ion.ucl.ac.uk/spm/). The principal eigenvariate (time series) was extracted for each ROI mask image and each subject. These signals were then averaged across all of the 2-back blocks and across all of the 0-back blocks separately.

### Network representation of ROI dependencies

In order to create network visualization we used the pair-wise dependency connectivity matrix. First we normalized the dependencies’ coefficients by using a Fisher Z transformation. Then a two-tailed t statistic was computed to compare the two conditions (e.g. left vs. right hand/foot movements). In order to construct a weighted adjacency matrix we applied a threshold connecting all pair-wise ROIs with dependencies that were significantly different between the two conditions (p < 0.05 level). This procedure allows for a simple graph visualization of the differences between the conditions across all subjects. The brain visualization of the graph was conducted with the BrainNet Viewer (Xia *et al*. 2013, http://www.nitrc.org/projects/bnv/)^58^.

In addition, to create a visualization of the influences of nodes on a particular edge (i.e. upon the correlations between two specific ROIs) we used the correlation influence d values (Equation 3). We first normalized the correlation influences coefficients by using a Fisher Z transformation. Then a two-tailed t statistic was computed to compare the two conditions (e.g. left vs. right hand/foot movements). All ROIs with correlation influence that were significantly different between the two conditions (p < 0.05 level) were plotted. This procedure allows for a simple visualization of the influences on a specific edge in the network between conditions across all subjects.

### Context related analysis

The D_EP_NA was computed for each subject and condition (i.e. left and right hand/foot movements in the motor task, or 2-back and 0-back in the working memory task) resulting in an *‘Influencing Degree’* and *‘Influenced Degree’* of each network region for each condition per subject. Next, a t-test between the two conditions was conducted across subjects and the results were corrected for multiple comparisons using false discovery rate (FDR)^59^ to estimate statistical significance. The FDR correction threshold was set to 0.05.

## Additional Information

**How to cite this article**: Jacob, Y. *et al*. Dependency Network Analysis (D_EP_NA) Reveals Context Related Influence of Brain Network Nodes. *Sci. Rep.* **6**, 27444; doi: 10.1038/srep27444 (2016).

## Supplementary Material

Supplementary Information

## Figures and Tables

**Table 1:** Left vs. Right Hand/Foot movement conditions T-test results.

**ROI**	**Hemisphere**	**Influencing Degree**	**Influenced Degree**
Precentral gyrus	L	**t** = **−4.59 p** = 1.28e-05*	t = 0.52 p = 0.60
	R	**t** = **5.84 p** = 6.67e-08*	t=−0.014 p = 0.99
SMA	L	t = −0.66 p = 0.51	t = 0.56 p = 0.58
	R	t = 0.35 p = 0.72	t = 0.17 p = 0.87
Thalamus	L	t = −2.51 p = 0.014	t = −0.20 p = 0.84
	R	t = 1.59 p = 0.11	t = 0.98 p = 0.33
Cerebellum	L	t = 0.50 p = 0.62	t = 1.22 p = 0.23
	R	t = −0.078 p = 0.94	t = 0.17 p = 0.86

A t-test comparison in each network region’s ‘Influencing Degree’ between the two conditions (i.e. Left vs. Right hand/foot movement) conducted across all 100 subjects.

*p < 0.05 FDR corrected.

**Table 2:** 2-back vs. 0-back T-test results.

**ROI**	**Influencing Degree**	**Influenced Degree**
L VLPFC	**t** = **7.10 p** = 1.9e-10 *	**t** = **6.11 p** = 1.98e-08*
R VLPFC	**t** = **6.59 p** = 2.11e-09*	**t** = **6.62 p** = 1.83e-09 *
R IPL	**t** = **5.94 p** = 4.28e-08*	**t** = **5.28 p** = 7.55e-07 *
R Premotor	**t** = **4.95 p** = 3.05e-06*	**t** = **5.10 p** = 1.63e-06*
R DLPFC	**t** = **4.80 p** = 5.64e-06*	**t** = **5.13 p** = 1.44e-06 *
Medial cerebellum	**t** = **3.89 p** = 0.00018 *	**t** = **5.06 p** = 1.97e-06 *
L IPL	**t** = **3.79 p** = 0.00026*	**t** = **4.75 p** = 6.84e-06*
L Frontal Pole	**t** = **2.98 p** = 0.0036*	**t** = **3.72 p** = **0.00033** *
L Premotor	**t** = **2.90 p** = 0.0046*	**t** = **3.92 p** = **0.00016** *
Medial posterior parietal	**t = 1.94 p = 0.055**	**t** = **2.94 p** = 0.0041*
R SMA	**t = 1.46 p = 0.15**	**t** = **3.84 p** = **0.00022** *
L DLPFC	**t = −1.26 p = 0.21**	**t** = **2.63 p** = 0.0097*

A t-test comparison in each network region’s influence degrees between the two conditions (i.e. 2-back and 0-back) conducted across all 100 subjects. *p < 0.05 FDR corrected.
