# Supplementary material for: Dependency Network Analysis (DEPNA) Reveals Context Related Influence of Brain Network Nodes
Source: Sci Rep. 2016 Jun 7;6:27444. doi: 10.1038/srep27444 (PMC4895213; doi:10.1038/srep27444)
Supplement: Supplementary Information [file srep27444-s1.pdf]

Supplementary Information

Dependency Network Analysis ($D_{EP}NA$) Reveals Context Related Influence of Brain Network Nodes

Yael Jacob, Yonatan Winetraub, Gal Raz, Eti Ben-Simon, Hadas Okon-Singer,

Keren Rosenberg-Katz, Talma Hendler, Eshel Ben Jacob

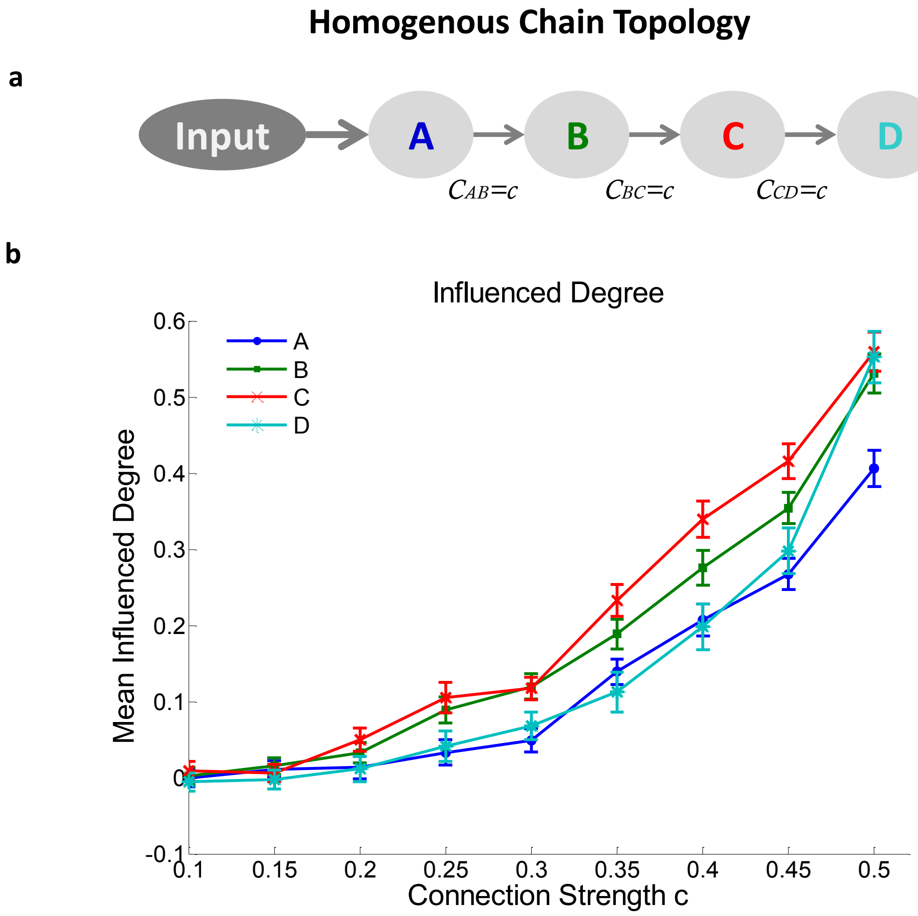

Figure S1. Simulated simple chain topology with homogenous connection strength 'Influenced Degree' results. (a) Illustration of the simple chain network topology. The arrows indicate the directed connection between the regions (i.e. the flow of information from one region to another), where c indicates the connection strength. Here we simulated a homologous network in which the connection strength between all regions is the same, while we adjusted it to range from a weak connection strength of 0.1 to a strong connection strength of 0.5. For each network connection strength the $D_{EP}NA$ was conducted on 200 randomized simulated BOLD signals (20 subjects x 10 trials). The DpNA conducted on the simulated data results of the 'Influenced Degree' **(b).** The $D_{EP}NA$, as expected, had correctly captured the network's flow of information (i.e. the hierarchy of influence) for the first three regions in the chain, whereas, the forth region did not obtain the highest 'Influenced Degree' as would have been expected. The $D_{EP}NA$ did not successfully captured the forth region's dependencies on account of the signal decay in time and thus it's poor SNR.

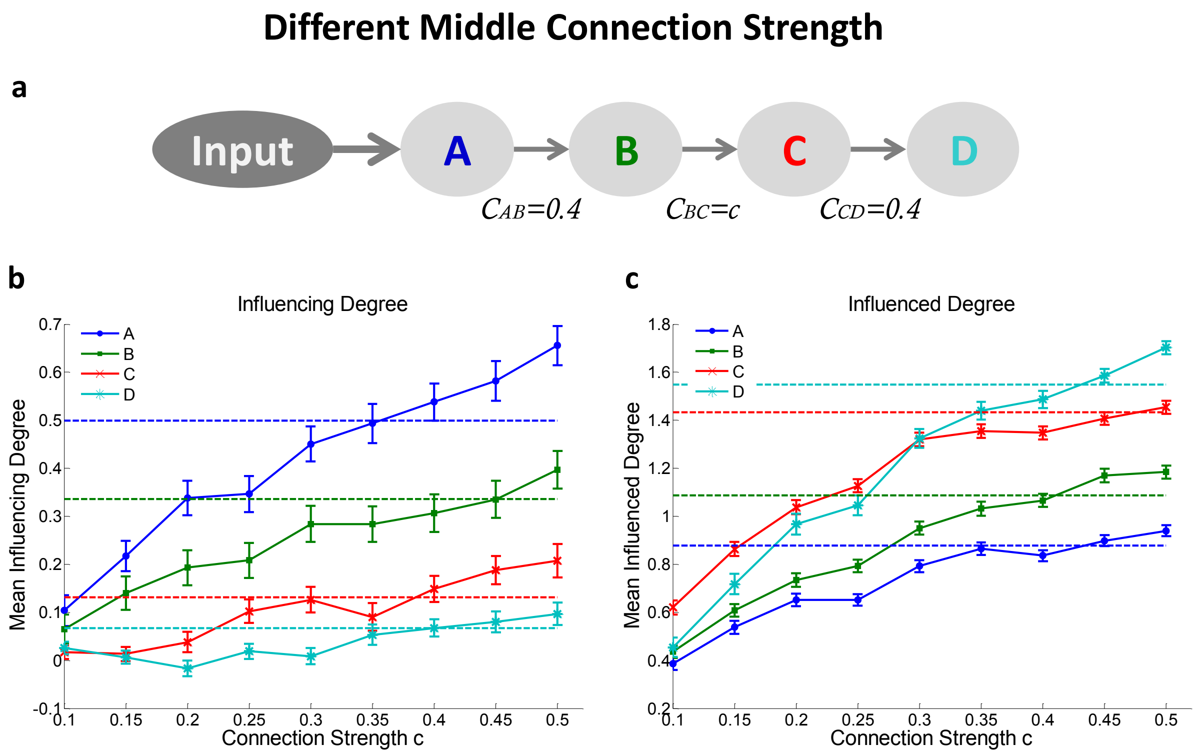

Figure S2. Simulated simple chain topology with different middle connection strength. (a) Illustration of the simple chain network topology. The arrows indicate the directed connection between the regions (i.e. the flow of information from one region to another), where c indicates the connection strength. Here we simulated a network in which the connection strength between all regions is the same and set to 0.4, while we adjusted only the middle connection (between regions B and C) to range from a weak connection strength of 0.1 to a strong connection strength of 0.5. For each network connection strength D_{EP}NA was conducted on 200 randomized simulated BOLD signals (20 subjects x 10 trials). D_{EP}NA conducted on the simulated data results of 'Influencing Degree' **(b)** and 'Influenced Degree' **(c).** For comparison, the dashed lines indicate each region's influencing or influenced degree as resulted in the homogenous chain simulation where all connections were set to 0.4. These results demonstrate that strengthening the middle connection increases the 'Influencing Degree' of upstream regions in the chain, whereas, only a minor effect is found on downstream regions. The 'Influenced Degree' demonstrates the inverse effect of higher 'Influenced Degree' for the downstream regions on the network.

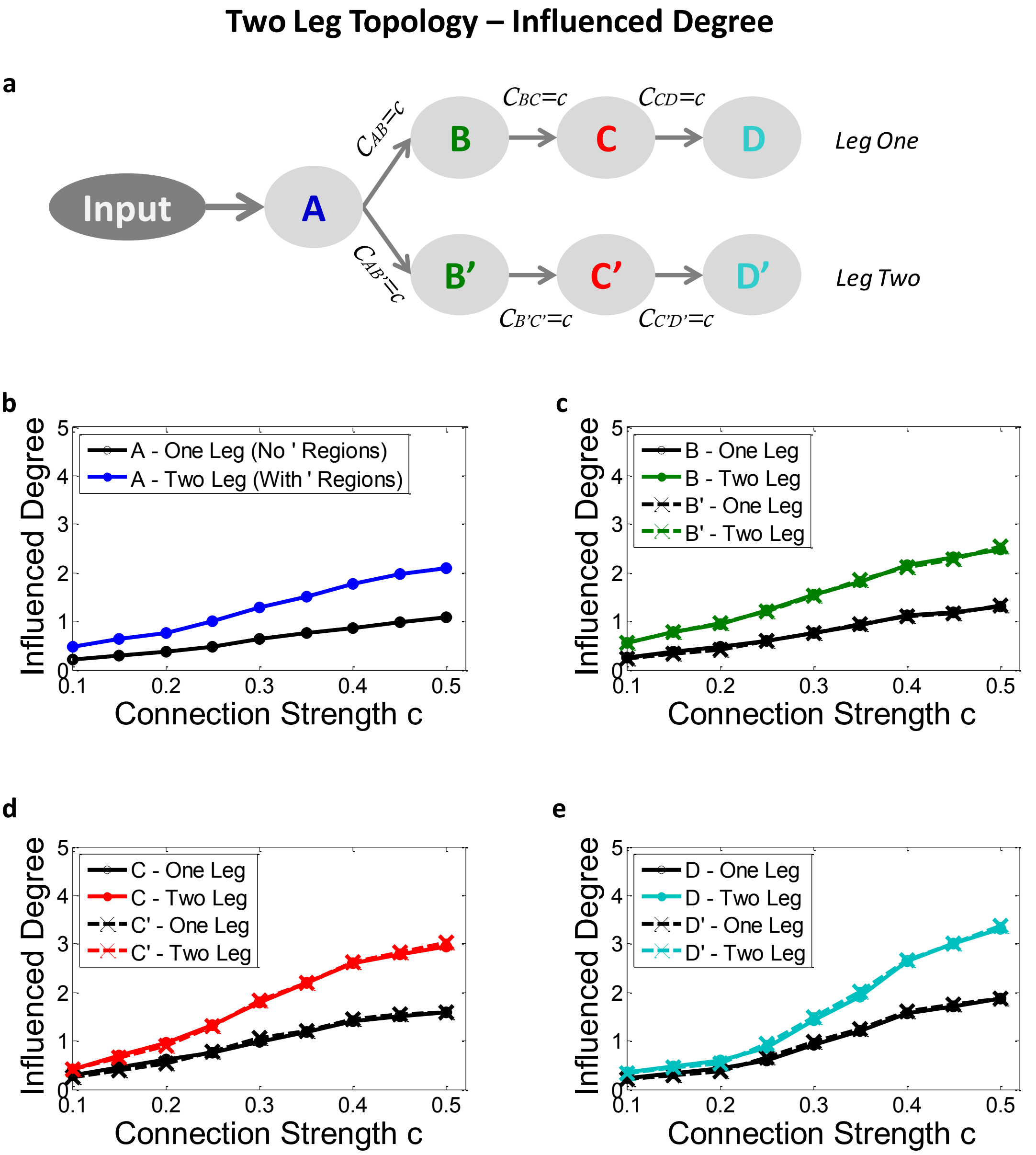

Figure S3. Simulated two-leg topology 'Influenced Degree' results. (A) Illustration of the two-leg network topology (as in Fig.3). Here we simulated a network where a single region influences two separate regions, creating two alternative paths. The D_{EP}NA simulation results, showing the 'Influenced Degree' of the network regions in the two-leg versus one-leg (simple chain topology as in Fig.2) scenario **(B-E)**. The last regions in the path (D and D') have the highest 'Influenced Degree', whereas the first region A has the lowest values.

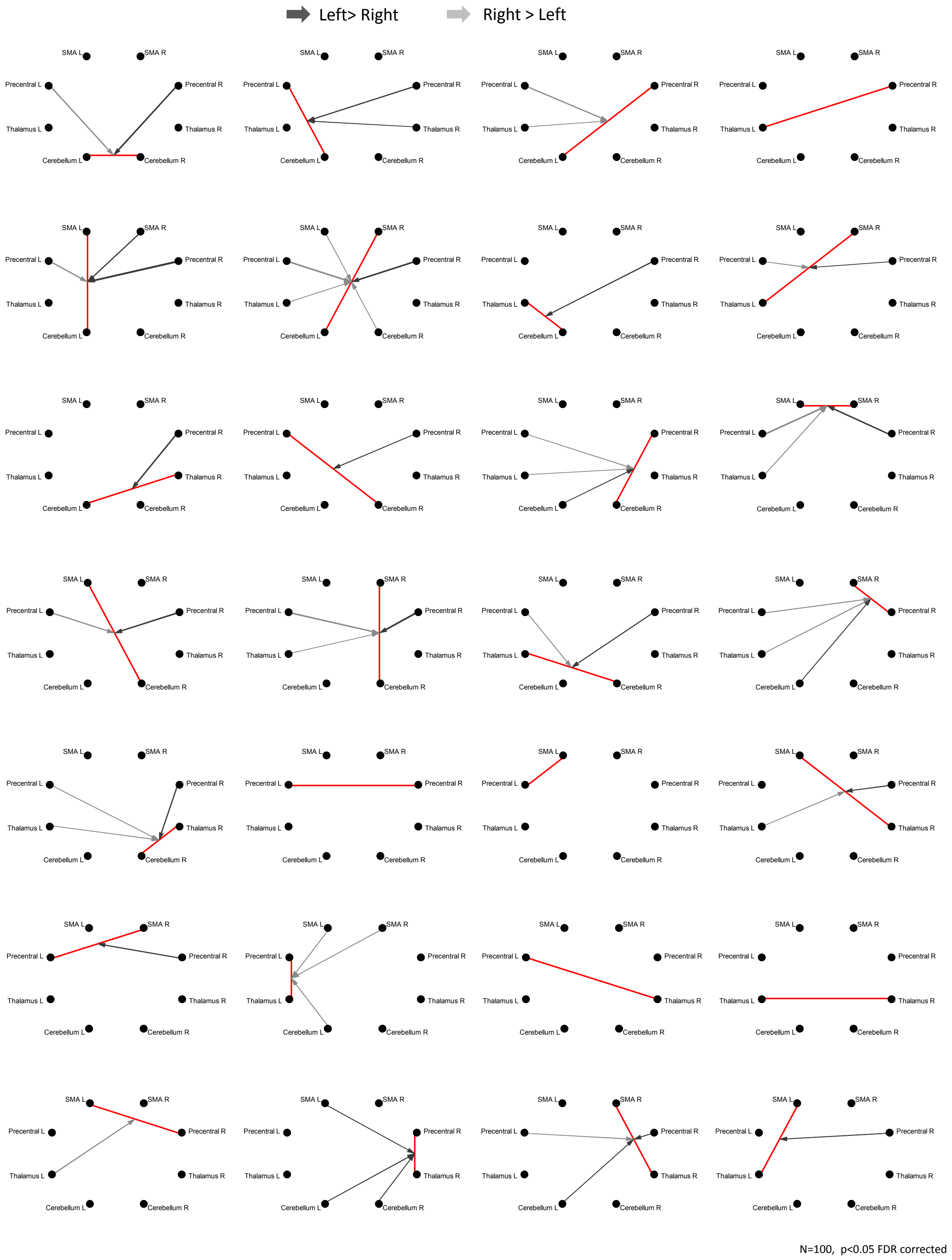

Figure S4. Visualization of nodes' influence on particular edges. Here we demonstrate the $D_{EP}NA$ capability to capture the influences on a particular edge in the network (i.e. upon the correlations between two specific ROIs). Each graph represents the influences on a different network edge where the particular edge is represented by a red line (total of 28 edges for a network consisting of 8 nodes). All ROIs with correlation influence d (see Figure 1a in results section or equation 3 in method section) that were found significantly different between conditions at the q<0.05 FDR corrected level are plotted as edges.

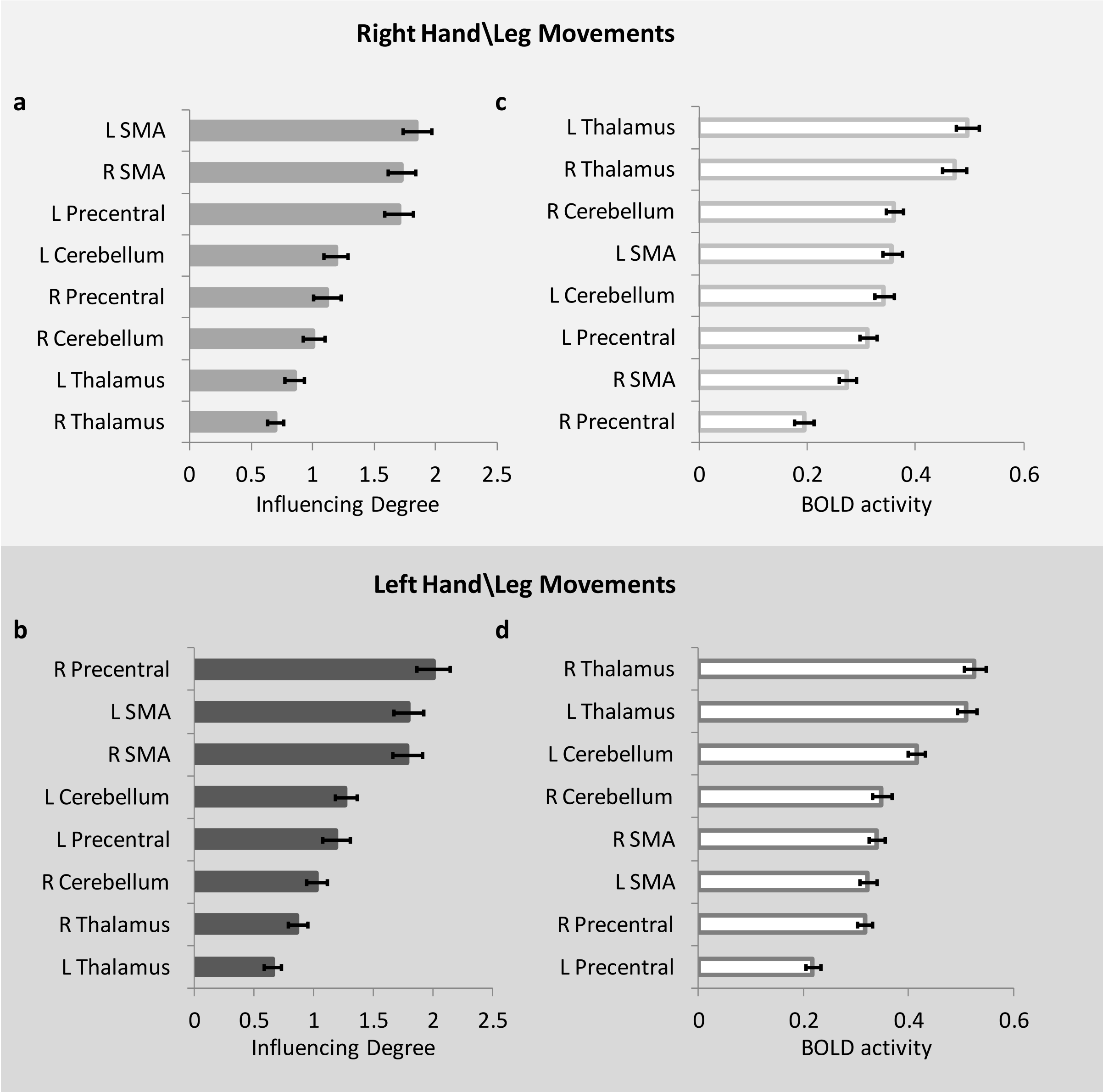

Figure S5. Network hierarchies outlined by BOLD activation magnitude and by D_{EP}NA. The motor network hierarchy as obtained by the D_{EP}NA *'Influencing Degree'* during right hand\leg movement condition **(a)** and during left hand\leg movement condition **(b)**. The motor network hierarchy as obtained by the BOLD activation magnitude during right hand\leg movement condition **(c)** and during left hand\leg movement condition **(d)**. The BOLD fMRI signal magnitude was computed as the percent signal change pick during each condition in each network region. These results indicate that D_{EP}NA reveals information which is not provided by standard analysis of BOLD activation; information about connectivity in the network and about the information flow hierarchies.
